# Supplementary figures and images for: Virus-infection in cochlear supporting cells induces audiosensory receptor hair cell death by TRAIL-induced necroptosis
Source: PLoS One. 2021 Nov 29;16(11):e0260443. doi: 10.1371/journal.pone.0260443 (PMC8629241; doi:10.1371/journal.pone.0260443)

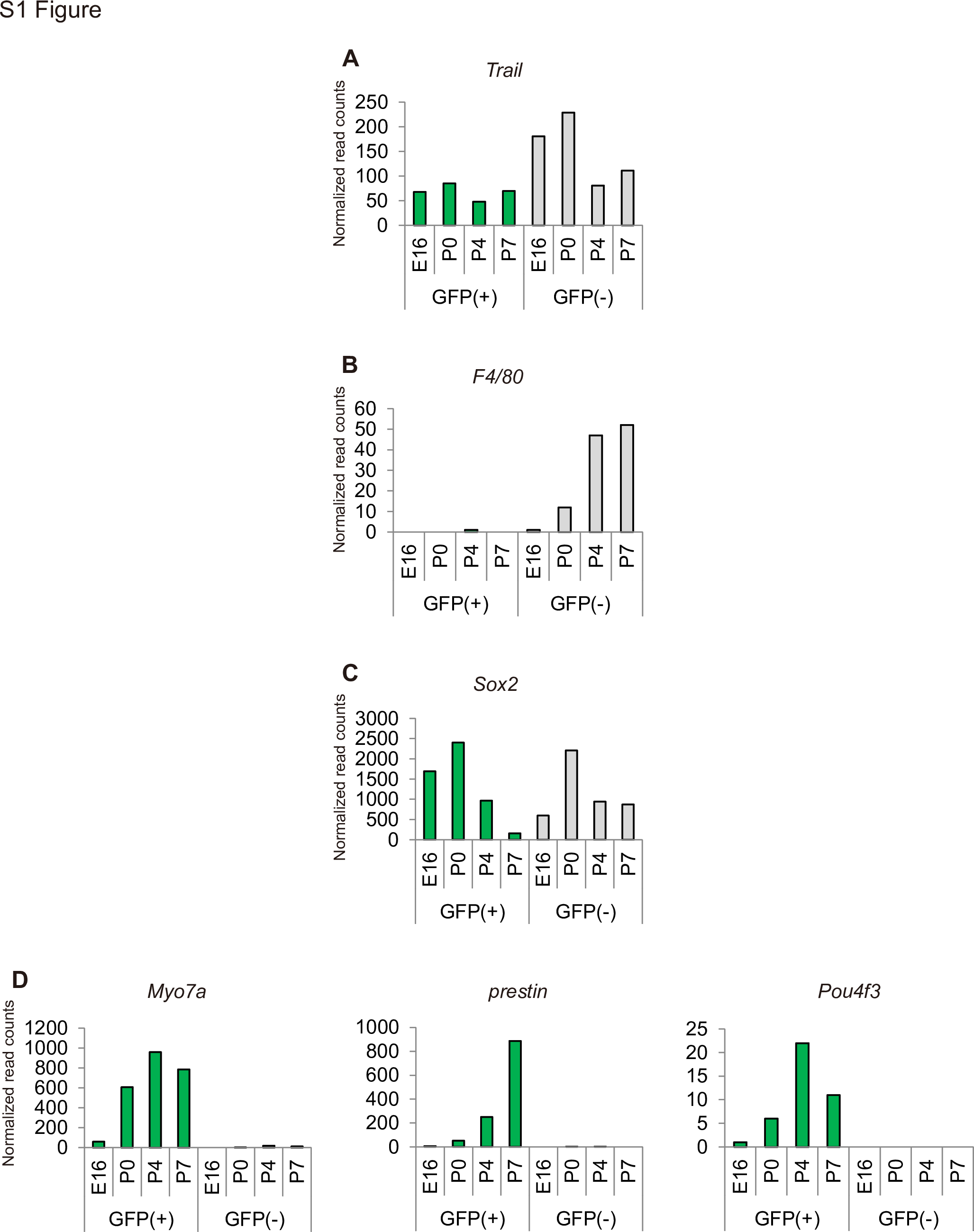

Supplement: S1 Fig — Expression changes of genes correlated with HCs and SCs during development. (A–D) Shared Harvard Inner-ear Laboratory Database (SHIELD; https://shield.hms.harvard.edu/index.html) is a resource for RNAseq datasets from HCs (GFP-positive cells) and their surrounding cells including SCs (GFP-negative cells) at E16, P0, P4, and P7. Here, we extracted Trail (A), F4/80 (B), Sox2 (C), and Myo7a, prestin, and Pou4f3 (D) from this database. (A) Trail was expressed in SC fractions higher than HC fractions, especially at E16 and P0. (B) Macrophage marker F4/80 was expressed in SC fractions. (C) SC marker Sox2 was expressed in not only SC fractions, but also HC fractions during the embryonic stage, but the SC fractions maintained Sox2 expression, whereas the HC fractions did not after the postnatal stage. (D) HC markers Myo7a, prestin, and Pou4f3 were expressed in HC fractions. (TIF) [file pone.0260443.s001.tif]

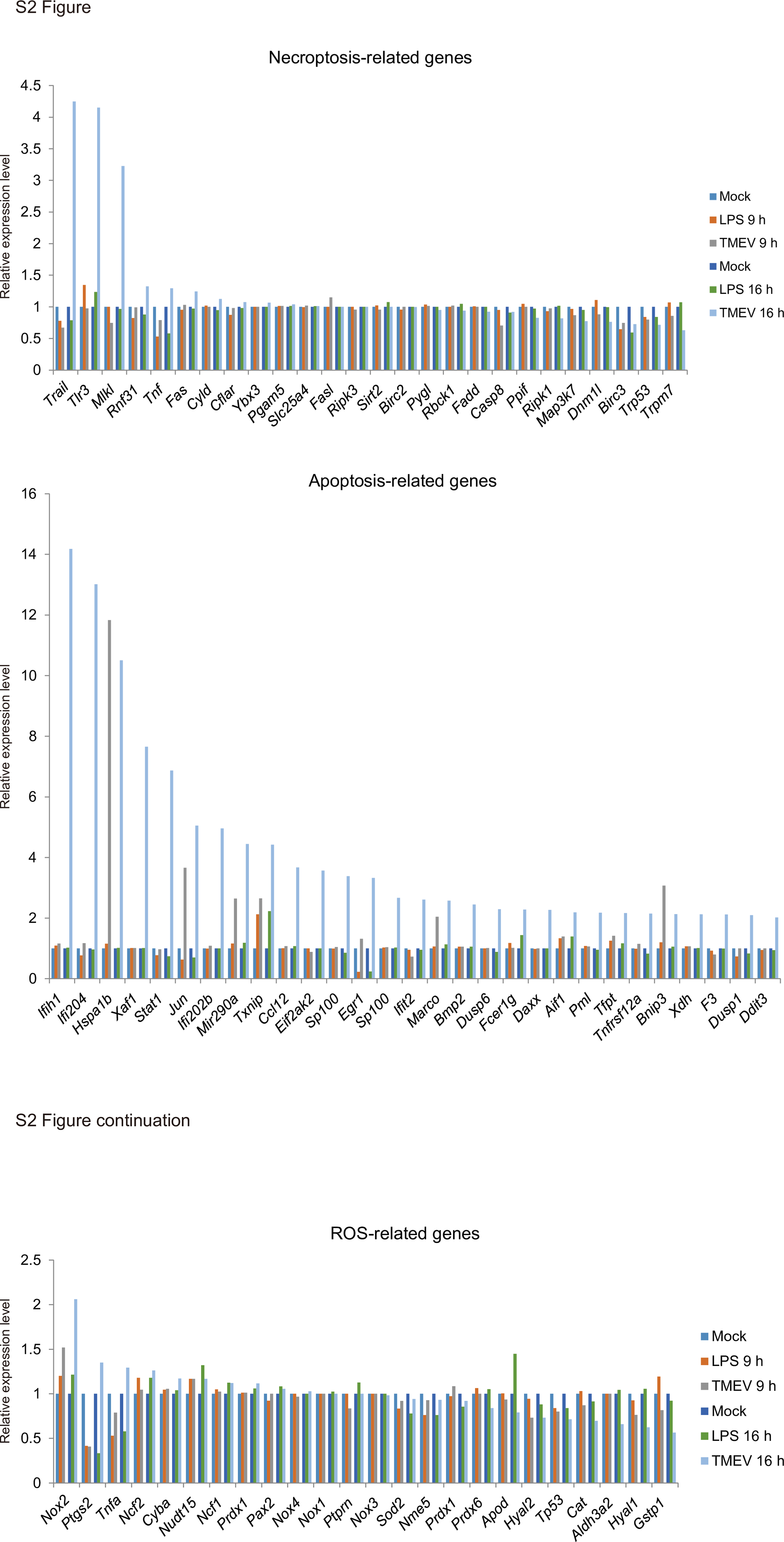

Supplement: S2 Fig — Gene Ontology analysis of microarray data showing upregulation of necroptosis- and apoptosis-related genes by TMEV infection. We performed microarray analysis of mock cochlear sensory epithelia, LPS-treated cochlear sensory epithelia (9 and 16 h), and TMEV-infected cochlear sensory epithelia (9 and 16 h) and then examined necroptosis-, apoptosis- and ROS-related genes by Gene Ontology analysis. Among necroptosis-related genes, Trail, Tlr3, and Mlkl were upregulated in TMEV-infected cochlear sensory epithelia at 16 h compared with mock- and LPS-treated cochlear sensory epithelia. Apoptosis-related genes, such as Stat1 and Jun, were upregulated in TMEV-infected cochlear sensory epithelia, especially at 16 h compared with mock- and LPS-treated cochlear sensory epithelia. However, ROS-related genes were not upregulated, except for Nox2, in TMEV-infected cochlear sensory epithelia compared with mock- and LPS-treated cochlear sensory epithelia. (TIF) [file pone.0260443.s002.tif]
